# Comparison of NK cell subsets, receptors and functions induced by radiofrequency ablation and microwave ablation in HBV-associated primary hepatocellular carcinoma

**DOI:** 10.3389/fonc.2023.1048049

**Published:** 2023-05-02

**Authors:** Hai-Yan Wang, Xiong-Wei Cui, Yong-Hong Zhang, Yu Chen, Ning-Ning Lu, Shou-Peng Sheng, Wen-Feng Gao, Xiao-Zheng Yang, Zhong-Ping Duan

**Affiliations:** Beijing Youan Hospital, Capital Medical University, Beijing, China

**Keywords:** thermal ablation, natural killer cells, hepatocellular carcinoma, radio frequency ablation, microwave ablation

## Abstract

**Background:**

Topical therapy has been shown to induce an immune response in patients with hepatocellular carcinoma (HCC). In this study, a prospective parallel group control experiment was conducted to compare the differences between radiofrequency ablation and microwave ablation in inducing the immune regulation of NK cells.

**Methods:**

Sixty patients with clinically and pathologically confirmed hepatitis B-associated hepatocellular carcinoma (HCC) were selected for thermal ablation. Patients were randomly assigned into the MWA group (n = 30) and the RFA group (n = 30). Patient’s peripheral blood was isolated on days D0, D7, and month M1. NK cell subsets, receptors, and killing function were detected by flow cytometry and LDH. Student t test and rank sum test were used to compare the statistical differences between the RFA (radio frequency) and MWA (microwave) groups. The Kaplan-Meier curve and log-rank test were used to calculate the difference between the two survival curves.

**Results:**

Comparison of the frequency of CD3-CD56+ and CD3-CD56+CD16+ in NK cells between the RFA and WMA groups showed that there was no difference in the D0, D7, M1, D7-D0, M1-D0, and M1-D7 groups. The changes of the inhibitory NK cell receptor CD159A were significantly different at D7 (P<0.05). CD107a were compared between the RFA and WMA groups, indicating that CD107a changes induced by NK cells were significantly different at D7-D0 (P<0.05). Comparison of NK cell lysis activity of target K562 cells between the RFA and WMA groups showed that there was no difference at D0, D7, D7-D0. There was no difference in recurrence-free survival (RFS) between the RFA and WMA groups (P=0.11).

**Conclusions:**

The difference between MWA and RFA-induced NK cell changes was mainly manifested in the inhibitory receptors CD159a and CD107a 1 week after surgery, with microwave-induced changes being more severe. Comparison of the NK cell lysis activity of the target K562 cells between the RFA and WMA groups showed that there was no difference in D0, D7, D7- D0. Survival analysis showed that these differences did not affect the recurrence-free survival (RFS) in the two groups.

## Introduction

Hepatocellular carcinoma (HCC) is currently the seventh most common cancer and the second leading cause of cancer-related deaths worldwide (1). Although up to 60% of HCC patients in developed countries are eligible for treatment such as surgery or ablation at the time of diagnosis (2, 3), tumor recurrence and long-term survival remain unresolved issues. Image-guided ablation has gained acceptance in the treatment of HCC in recent years due to its safety and efficacy. Among HCC types, percutaneous radiofrequency ablation (RFA) has become the standard treatment for unresectable early-stage HCC and is even competitive with surgery for single nodules less than 3 cm in diameter (4, 5). However, survival has been found to be compromised by significant rates of local and distant recurrence (6, 7). Among the newer ablation techniques, microwave ablation (MWA) has played a key role in the thermal ablation of HCC as a valuable alternative to RFA. Compared with other thermal ablation techniques, the main advantages of MWA technology are higher temperature within the tumor, greater tumor ablation, faster melting time, and improved convection shape, reducing the risk of endothermic effects (3). Although MWA appears superior to RFA, neither prospective nor retrospective studies have successfully demonstrated a significant difference between the two ablation modalities (4, 8, 9).

Percutaneous thermal ablation induces immunomodulation in patients with hepatocellular carcinoma. In the case of RFA and MWA, heat delivered to the cancerous tissue triggers cell death through a process called necrosis, which leads to the uncontrolled release of cellular components into the extracellular space and triggers an inflammatory response. Initially, innate immune cells respond rapidly and infiltrate into the tissue site, subsequently activating adaptive immune cells (10, 11). This process also triggers antitumor responses, as these tumor-specific antigens are exposed to components of the entire immune system.

Because RFA is the most commonly used thermal ablation technique, several studies have examined the effects of RFA on the immune system. Heat shock protein (Hsp) is a chaperone involved in several oncogenic processes that are highly expressed either inside or outside cancer cells. Hsp-70 is released in large amounts in serum after RFA and appears to be an activator of innate and adaptive immune responses, as well as activating antigenic peptides and promoting danger signals. The release of HSP-70 may lead to local inflammation and activation of antigen-presenting cells in the tumor area, which in turn stimulates the antitumor response (12). Another study showed that RFA enhances cross-presentation by antigen-presenting cells. Using a mouse model, Dromi et al. found that RFA led to increased infiltration of dendritic cells (DC) and significantly increased CD4+ and CD8+ T-cell responses in tumors (13). Mizukoshi et al. found that TAA -specific T cells were significantly increased after RFA and most of them were CD8+ T cells (14).

A recent study reported that innate cells may play an important role in the immune dynamics of RFA in HCC, with immune regulation starting very early. Indeed, DC and natural killer (NK) cells are recruited to the tumor early after RFA to enhance the antitumor response. NK cells lyse target cells by balancing activating and suppressing receptors. Shortly after RFA, a high number of NK cells expressing the activated receptor NKp30 was associated with a lower rate of tumor recurrence (15).

The immunomodulatory role of MWA has been less studied than that of RFA, probably because MWA is a less common and newer ablation technique. In RFA, cell debris, such as HSP-70 is released during heating. However, Ahmad et al. described lower serum levels of these proteins after hepatic MWA compared with RFA in a rat model. The proinflammatory cytokines IL-1β and IL-6 were also increased in the circulation after MWA, suggesting the induction of an inflammatory response. Although circulating IL-1β levels were not significantly different between RFA and MWA, the increase in IL-6 in the blood of MWA-treated rats was significantly less than that of RFA-treated rats. These results suggest that MWA may elicit a lower inflammatory response than RFA in rats (16).

Leuchte K analyzed tumor-specific immune responses in relation to T-cell responses and disease progression in peripheral blood of HCC patients after microwave ablation (17). MWA had only moderate effects on subsets of circulating immune cells, whereas fluorescence dot analysis of specific T-cell responses to seven tumor-associated antigens (TTAs) revealed incipient or enhanced tumor-specific immune responses in 30% of patients. Digital image analysis of immunohistochemically stained HCC profile samples (n = 18) from patients undergoing combined MWA and excision showed that patients with high T-cell abundance had better disease-free survival with thermal ablation (37.4 vs. 13.1 months) (17). MWA also promotes infiltration of immune cells into liver tissue, as shown in a study of liver biopsies from patients before and after MWA. Among the infiltrating intrahepatic immune cells, mainly NK cells and macrophages were observed, as well as a large number of T cells (18).

All of the above observations indicate that radiofrequency and microwave ablation techniques can induce immunomodulation. Nevertheless, there is no comparison of which thermal ablation technique is more effective in inducing immunomodulation. In this study, a prospective controlled parallel group experiment was performed to compare the dynamic changes of NK cell subsets, receptors, and functions induced by RFA and MWA, and to compare the differences between RFA and MWA in inducing NK cell immunoregulation and its effects on disease-free survival.

## Materials and methods

### Ethical statement

The selected patients were admitted to Beijing Youan Hospital, Capital Medical University, from January 2020 to April 2021. They provided written informed consent to participate in the study. This study and other related experiments were approved by the Hospital Research Ethics Committee of Beijing You (No (2019).073) and written informed consent was obtained in accordance with the Declaration of Helsinki. The study was conducted in accordance with the approved guidelines and regulations.

### Study population

As a prospective controlled parallel group study, participating patients were enrolled in Beijing You an Hospital Affiliated with Capital Medical College from January 2020 to April 2021. According to the Standard for the Diagnosis and Treatment of Primary Liver Cancer (2019 Edition) of the Medical Administration and Medical Management Bureau of the National Health Commission, PRC, 60 patients with HBV-related primary liver cancer (HCC) were diagnosed clinically and pathologically. Patients with a single tumor diameter of ≤3 cm or no more than 2 tumor nodules, a maximum tumor diameter of ≤3 cm, and no vascular, biliary, or adjacent organ invasion or distant metastasis were classified as Child-Pugh grade A liver function. All cases were excluded from other hepatitis virus infections, autoimmune diseases, alcoholic liver diseases, etc. At the same time, 60 patients were randomly assigned to microwave ablation (MWA) group (n = 30) and radiofrequency ablation (RFA) group (n = 30). All patients were treated with thermal ablation (RFA or MWA) after enrolment. The study was approved by the ethics committee of Beijing Youan Hospital, and all patients signed an informed consent form.

### Study design

From January 8, 2020, to April 1, 2021, 60 patients were enrolled in the study and randomly assigned to the MWA group (n = 27) and the RFA group (n = 29). Three patients (4%) only partially received the assigned intervention and were excluded. Two of these patients received the assigned intervention (RFA) for early-stage HCC but did not receive the same intervention for later-stage metachronous tumors because of procedural errors. A third patient with bifocal HCC received a planned allocation intervention (MWA) for one tumor but did not undergo ablation for the second tumor. One additional patient (2%) was excluded from analysis because of a different diagnosis (intrahepatic cholangiocarcinoma). These patients were treated under conscious sedation and CT -guided thermal ablation. RFA was connected to a radiofrequency generator (model 1500 radiofrequency generator, RITA Medical System, Inc., a Mountain View, CA); MWA was connected to a microwave generator with ECO-100AL3 (MTC-3C Nanjing Viking Jiuzhou Medical Device R&D Center). Three radiologists (AR, AZ, and JS) with 10 years of experience in interventional oncology performed the ablation procedure. Tumor ablation was completed in a single session, with an ablation margin of 5-10 mm as the overall goal. At the end of the procedure, the ablated area was evaluated by extended CT. In a single treatment session for tumor ablation, an enamel margin of 5-10 mm was the goal. At the end of the procedure, the ablation area was observed using Enhanced CT. If the margin was < 5 mm, the margin was extended to ensure complete necrosis. Thermal ablation and posttreatment assessment were performed by two interventionalists (CXW and SB) with more than 10 years of clinical experience. The therapeutic efficacy of HCC met the efficacy evaluation criteria for solid tumors (m-RECIST standard) (19).

Radiological evaluation of the ablation process was performed one month after the intervention and then every three months, with follow-up until recurrence or termination of the study. The date of recurrence was determined by the time of tumor progression (TTP) when the first radiographic study showed definite recurrence. Sequential blood samples were collected from 27 HCC patients treated with MWA (before the intervention, day seven, and day 30) and 29 HCC patients treated with RFA (before the intervention, day seven, and day 30). One radiofrequency patient was lost during follow-up. The patient characteristics are summarised in **Table 1**. All patients gave written informed consent and the local ethics committee approved the study (No (2019).073).

**Table 1:** Clinical characteristics of baseline patients in the RFA and MWA groups.

		MWA	RFA	P-Value
n		27	28	
Gender	**female**	4 (14.8%)	8 (28.6%)	0.364
	**male**	23 (85.2%)	20 (71.4%)	
Age (y)		62.0 [54.5,66.0]	61.5 [56.8,66.2]	0.826
No. of nodules	**1**	25 (92.6%)	26 (92.9%)	1000
	**2**	2 (7.4%)	2 (7.1%)	
Cumulative diameter (mm)		210 [16.5,30.0]	20.0 [17.0,30.0]	0.643
ALT (IU/L)		270 [18.5,34.0]	21.5 [16.0,28.2]	0.18
AST (IU/L)		28.0 [20.5,39.0]	270 [210,30.0]	0.288
TBIL ( umolll)		16.2 [14.0,19.0]	17.6 [13.6,21.4]	0.643
AlB (giL)		40.4 [39.2,42.2]	39.8 [35.9,41.2]	0.203
PLT (10*9/L)		142.0 [96.0,216.0]	120.0 [84.0,146.2]	0.119
PTA%		87.0 [78.0,94.0]	82.0 [79.8,90.5]	0.619
AFP (ng/ml)		16.5 [2.3,70.0]	6.5 [3.1,23.6]	0.55
HBV-DNA (copies/ml)		42.0 [10.0,100.0]	10.0 [10.0,100.0]	0.436
FIB4		0.2 [0.1,0.4]	0.3 [0.2,0.4]	0.386
Child-Pugh	**5**	26 (96.3%)	24 (85.7%)	0.352
	**6**	I (3.7%)	4 (14.3%)	

The cumulative diameter is tumor size.

### Flow cytometric analysis

1) PBMC were separated by Ficoll-Hypaque density centrifugation, then stored at -80^0^C in a refrigerator.
2) Flow cytometry analysis.

After thawing, cells were incubated with specific surface and intracellular antibodies, and flow cytometry was performed using a BD Fortessa^®^ cytometer (BD Biosciences).

#### Recovery PBMC

**Figure 1:**
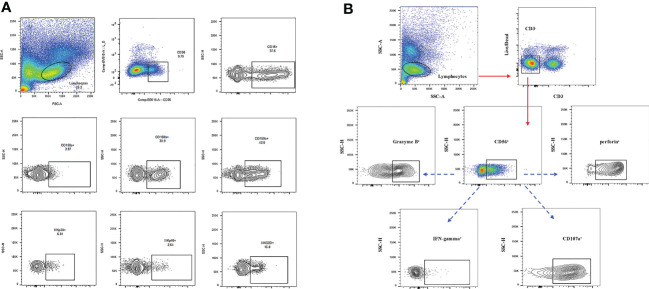
**(A)** The gating strategy used to identify NK cell subsets by flow cytometry and the frequencies of gated populations are shown for a representative HC. Lymphocytes were gated according to forward and side scatter dot plots. Single cells were gated according to forward height and side scatter forward area. Dead cells were excluded by staining with Live/Dead fixable viability stain 510. NK cells were defined within the CD3-gate on the basis of the expression of CD16 and CD56. **(B)** Cell surface cd3-apc-h7, cd56-bb515, and cd16-pe staining were performed first. After washing at 4°C for 20 mins, 2mL PBS was added and 600g cells were immersed for 3 mins to discard the supernatant. Then the cells were exploded and be product solution was added for 20 mins at 4 °C. After washing, IFN-γ-BV711, Perforin-AP647, Granzyme B-BV421, and CD107A-PE-CY7 were added and they were incubated at 4°C for 20 mins without light. After washing once with the broken membrane detergent, the machine was used for detection.

Tube.1: The following fluorescently labelled antibodies were added: Anti-cd3-apc-h7:1.25μl, anti-CD56-BB515:5 μL, anti-CD16-PE:10 μL, NKp46 - PE - cy7:3 mu l, NKp30 - BV421:3, mu l CD158a - APC: 2.5 mu, l CD158b - BV785: 1.25μl, NKG2D-BV605:3 μL, and NKG2A(CD159a)-BB700:3 μL. Then, they were incubated at 4 °C for 20 mins in darkness and washed once with PBS. Cells were harvested by the CELLQuest software and analyzed. The gating strategy is shown in **Figure 1A**.

Tube 2: The cells were stained with CD3-APC-H7, CD56-BB515, and CD16-PE on the cell surface. After washing, the cells were reacted at 4 °C for 20 mins, then the supernatant was discarded by adding 2 ml PBS and 600g submerged CELLL for 3 mins. The cells were shot and the membrane-breaking solution was added for 20 mins at 4 °C. Cd107a-pe-cy7, IFN-γ-BV711, Perforin-APC, and Granzyme B-BV421 were added after washing with broken membrane solution and incubated at 4 °C for 20 mins in the dark. After washing with the broken membrane solution once, the cells were tested on the machine. The gate-setting strategy is shown in **Figure 1B**.

#### Cytolytic assay

NK cell activity was detected by the LDH release assay. The PBMCs were resuscitated, washed once, plated on a six-well plate, and incubated in a 37°C incubator overnight. The cells were collected, 70-mesh filtered, centrifuged, counted, and adjusted at 100 µl containing 5×105. K562 cells at 100 µl containing 5×103 cells (effective target ratio of 100:1) were placed in 96-well plates and incubated at 37°C overnight. Each specimen was tested in triplicate. The natural release control group and the maximum release control group of target cells were set simultaneously. Water (10 µl) was added to each natural release well, 10 µl lysate was added to each maximum release well, and the 37°C incubation lasted 45 min. The supernatant (50 µl) was transferred to a new 96-well plate, 50 µl of the reaction solution was added (600 µl assay buffer and 11.4 mL of substrate stock solution) to each well, reacted at room temperature for 30 min, and stopped with 50 µl of stop solution. The plates were read at 490 nm. The percentage of cytotoxicity was calculated using the following formula: cytotoxicity (%) = (test sample - low control)/(high control - low control) %.

### Data statistics

1. Python (Version3.6) software was used for statistical analysis after checking all original data.
2. Lilliefors-test was used to test whether the data at three-time points and the changes at any two-time points were in line with the normal distribution, as well as whether the data could meet the homogeneity of variance. For the normal distribution of data with homogeneity of variance, the student’s t-test was used to compare the statistical differences between the RFA and MWA groups. Otherwise, the rank sum test was used to compare the statistical differences between the RFA and MWA groups. P<0.05 was considered significant.
3. The recurrence-free survival (RFS) time was calculated from the first ablation treatment. The follow-up time was calculated from the start of the ablation treatment to the date of the last follow-up (2022-12-30). According to the treatment method, the patients were divided into RFA and MWA groups. Kaplan-Meier curves were drawn respectively. The log-rank test was used to calculate the difference between the two survival curves. P<0.05 was statistically significant. The median survival time was 384 days for MWA and 460 days for RFA.

## Results

### Clinical outcome results

We recruited 56 patients with HBV-related HCC whose liver function was Child A and BCLN grade A stage. Details of the subjects (who were not undergoing co-infections or other diseases) are summarised in **Table 1**. There were no differences in tumor characteristics between the two groups (RFA and MWA), particularly in tumor size and volume (**Table 1**).

**Figure 2:**
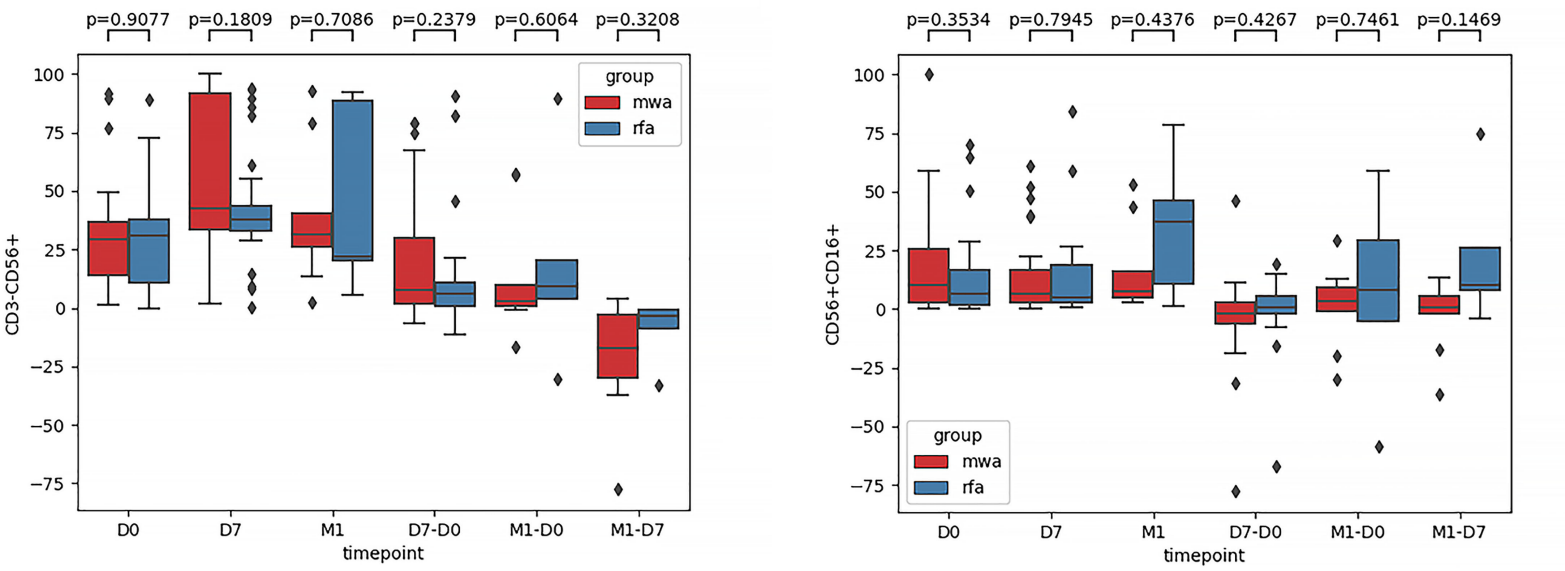
The frequencies of CD3-CD56+ and CD3-CD56+CD16+ in NK cells of the RFA group and WMA group were compared. It was suggested that D0 (P=0.91, P=0.35), D7 (P=0.18, P=0.79), M1 (P=0.71, P=0.44), D7-D0 (P=0.24, P=0.43), M1-D0 (P=0.61, P=0.75), M1, and D7 (P=0.32, P=0.15) in the two groups had no difference.

First, the frequency of CD3-CD56+ and CD3-CD56+CD16+ in the NK cells of the RFA and WMA groups was compared. We found that there was no difference between the two groups in D0 (P=0.91, P=0.35), D7 (P=0.18, P=0.79), M1 (P=0.71, P=0.44), D7-D0 (P=0.24, P=0.43), M1-D0 (P=0.61, P=0.75), M1-D7 (P=0.32, P=0.15) (**Figure 2**).

**Figure 3:**
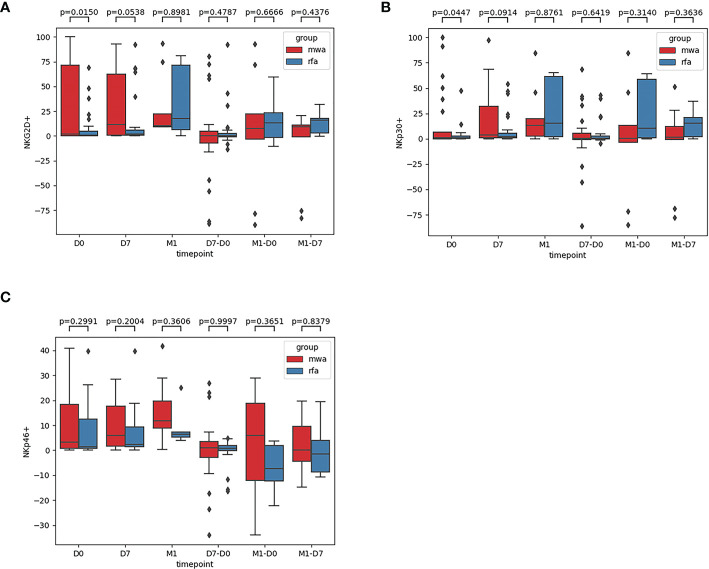
The frequencies of activated receptors NKG2D **(A)**, NKp30 **(B)**, and NKp46 **(C)** in the WMA group and RFA group were compared . The frequencies of activated receptors (NKG2D, NKp30, and NKp46) in D0(P=0.015, P=0.045, P=0.299), D7 (P= 0.054, P=0.091, P=0.2), M1 (P= 0.90, P=0.88, P=0.36), D7-D0 (P=0.48, P=0.64, P=0.99), M1-D0 (P=0.67, P=0.31, P=0.37), M1-D7 (P=0.44, P=0.36, P=0.84), indicating that in D0, There were differences in NK cell activation receptor NKG2D (P<0.05) **(A)** and NK cell activation receptor NKp30(P<0.05) **(B)**. At D7 NKG2D(P=0.054) **(A)**.

Second, comparisons of the frequencies of the activated receptors (NKG2D, NKp30, and NKp46) in the WMA and RFA groups were compared (**Figure 3**). Additionally, the frequencies of the activated receptors (NKG2D, NKp30, and NKp46) in D0 (P=0.015, P=0.045, P=0.299), D7 (P=0.054, P=0.091, P=0.2), and M1 (P=0.90, P=0.88, P=0.36) were also compared. D7-D0 (P=0.48, P=0.64, P=0.99), M1-D0 (P=0.67, P=0.31, P=0.37), and M1-D7 (P=0.44, P=0.36, P=0.84) indicated that, in D0, there were differences in NK cell activation receptor NKG2D (P<0.05) (**Figure 3A**) and NK cell activation receptor NKp30 (P<0.05) (**Figure 3B**). Moreover, differences in NK cell-activated receptors NKG2D in D7 were detected (P=0.054) (**Figure 3A**).

**Figure 4:**
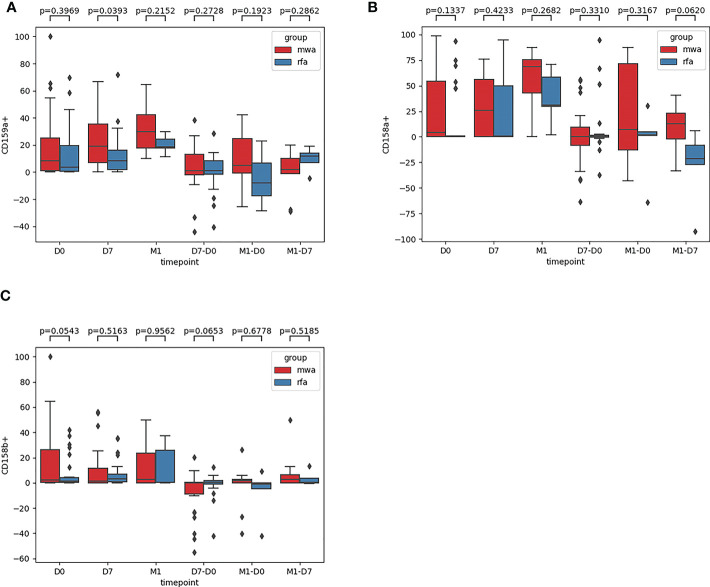
The frequencies of NK cell inhibitory receptors NKG2A [CD159A] **(A)**, CD158a **(B)**, CD158b **(C)** were compared between the RFA group and the MWA group. In D0(P=0.40,P= 0.13,P= 0.054), D7 (P= 0.04,P= 0.42,P=0.52), M1 (P = 0.22, P = 0.27, P = 0.96), D7 - D0 (P = 0.27, P = 0.33, P = 0.65), M1 - D0 (P = 0.19, P = 0.32, P = 0.68), M1, D7 (P = 0.29, P = 0.06, P = 0.52). The changes of NK cell inhibitory receptor CD159A(P<0.05) were significantly different between RFA group and MWA group one week after operation **(A)** (P<0.05).

Third, the frequencies of NK cell inhibitory receptors (NKG2A [CD159A], CD158a, CD158b) were compared between the RFA and MWA groups (**Figure 4**): in D0 (P=0.40, P= 0.13, P= 0.054), D7 (P= 0.04, P=0.42, P=0.52), M1 (P=0.22, P=0.27, P=0.96), D7-D0 (P=0.27, P=0.33, P=0.65), M1-D0 (P=0.19, P=0.32, P=0.68), M1- D7 (P=0.29, P=0.06, P=0.52). The changes of NK cell inhibitory receptor CD159A (P<0.05) were significantly different between the RFA and MWA groups one week after the surgery (**Figure 4A**) (P<0.05).

**Figure 5:**
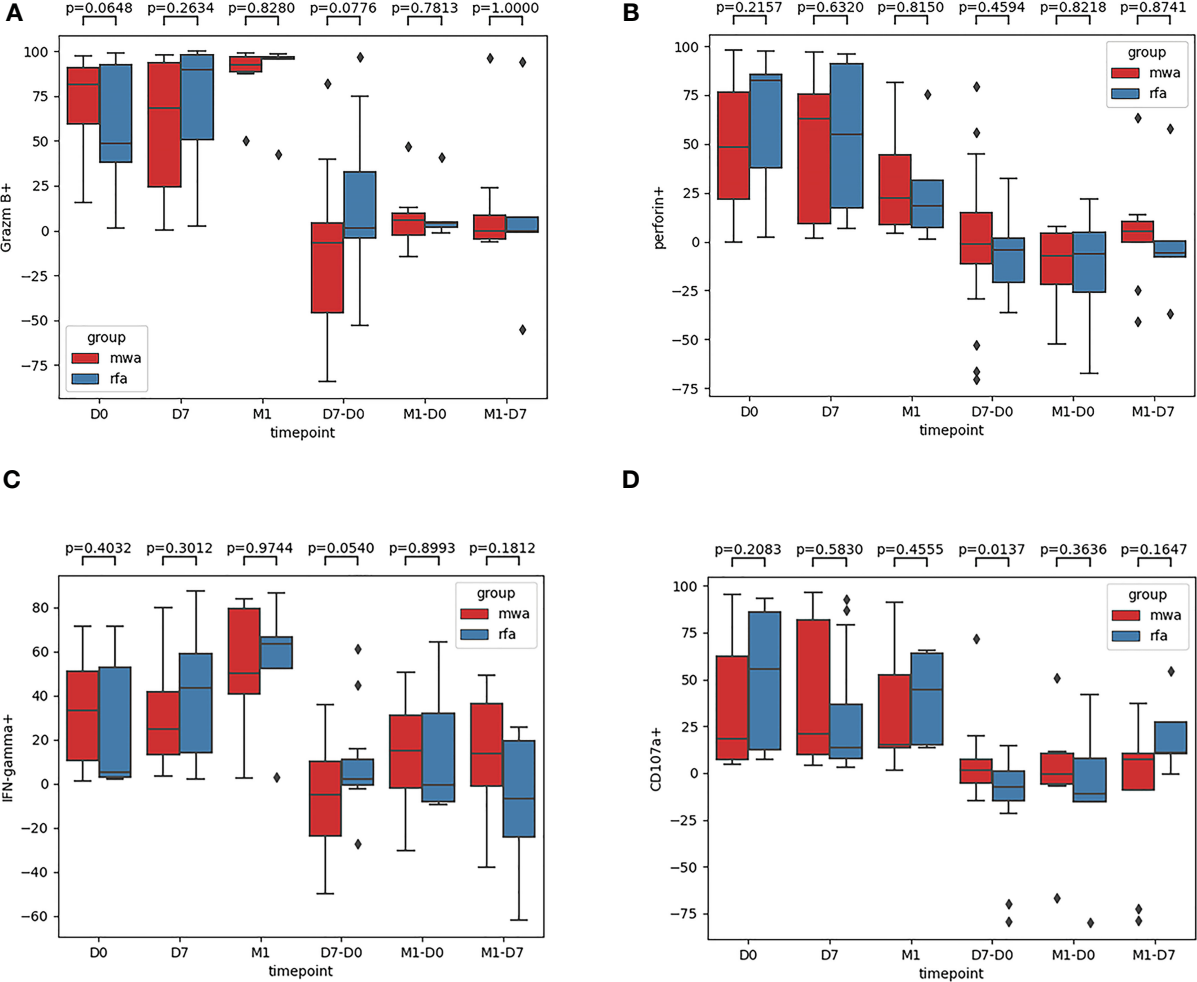
The frequencies of Granzyme B **(A)**, Perforin **(B)**, IFN-γ **(C)**, CD107a **(D)** were compared between the RFA group and the WMA group. In D0 (P=0.06,P=0.22,P=0.40,P=0.21), D7(P=0.26,P=0.63,P=0.30,P=0.58), M1(P=0.83,P=0.82,P= 0.97,P= 0.46), D7-D0 (P= 0.08,P= 0.46,P= 0.054,P= 0.012), M1-D0 (P= 0.78,P=0.82,P= 0.90, P=0.36), M1-D7(P=1,P=0.87,P=0.18,P=0.16), suggesting that NK cells were induced to produce CD107a was a significant difference between the RFA and WMA groups in D7-D0 (P<0.05) **(D)**. At D7-D0, IFN-γ(P=0.054)**(C)**.

Fourth, the frequencies of Granzyme B, Perforin, IFN-γ, and CD107a were compared between the RFA and WMA groups (**Figure 5**): in D0 (P=0.06, P=0.22, P=0.40, P=0.21), D7 (P=0.26, P=0.63, P=0.30, P=0.58), M1 (P=0.83, P=0.82, P=0.97, P= 0.46), D7-D0 (P=0.08, P=0.46, P=0.054, P=0.012), M1-D0 (P=0.78, P=0.82, P=0.90, P=0.36), and M1-D7 (P=1, P=0.87, P=0.18, P=0.16), suggesting that NK cells were induced to produce CD107a. There were significant differences between the RFA and WMA groups in D7-D0 (P<0.05) (**Figure 5D**). Moreover, in D7-D0, differences between the RFA group and the WMA group in inducing NK cells to produce IFN-γ were detected (P=0.054) (**Figure 5C**).

**Figure 6:**
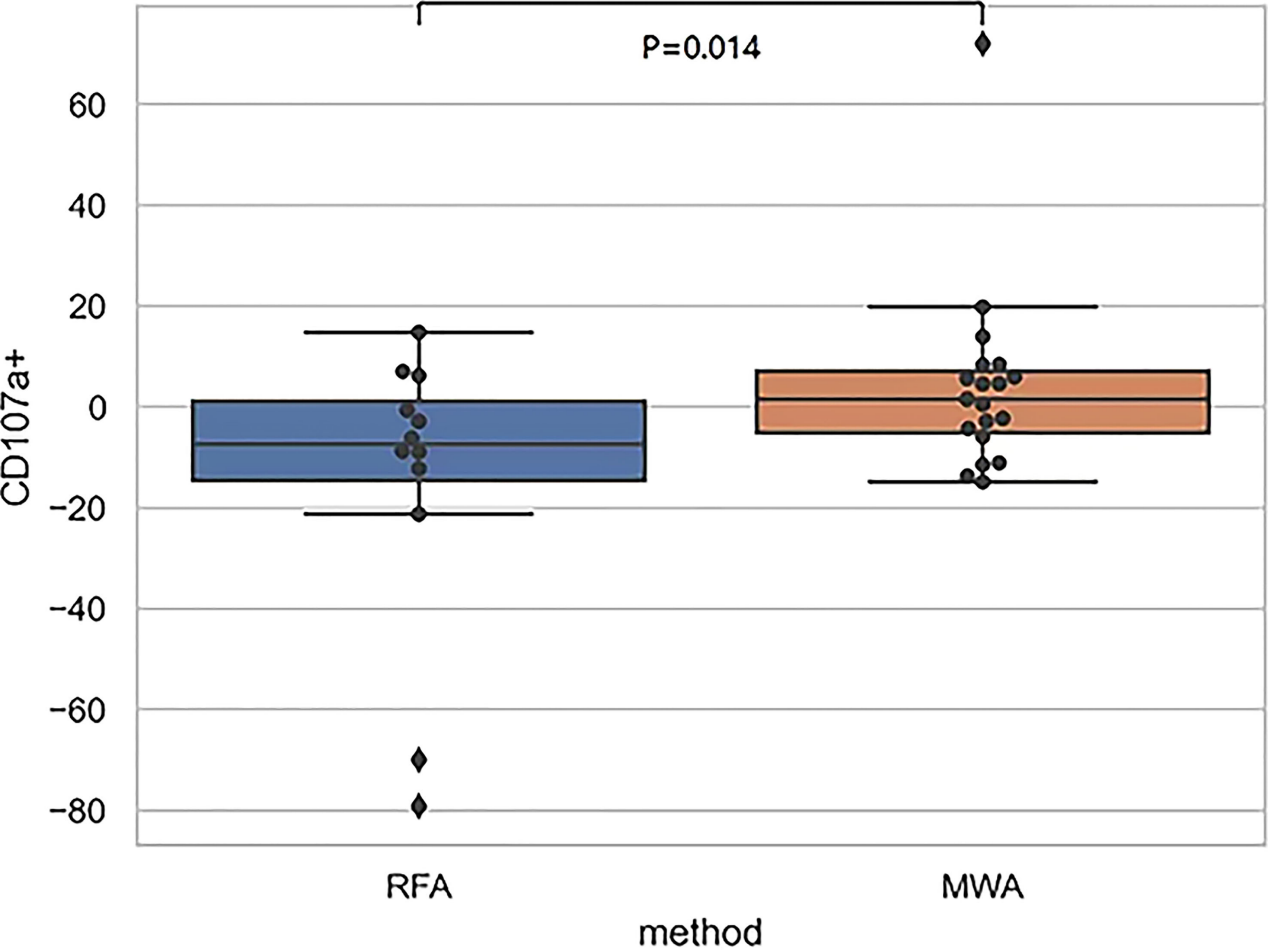
The comparison of CD107a between the RFA and WMA groups (D7-D0) suggested that there was a significant difference between the two groups in inducing CD107a production in NK cells at D7-D0 (P<0.05),microwave-induced changes were stronger.

**Figure 7:**
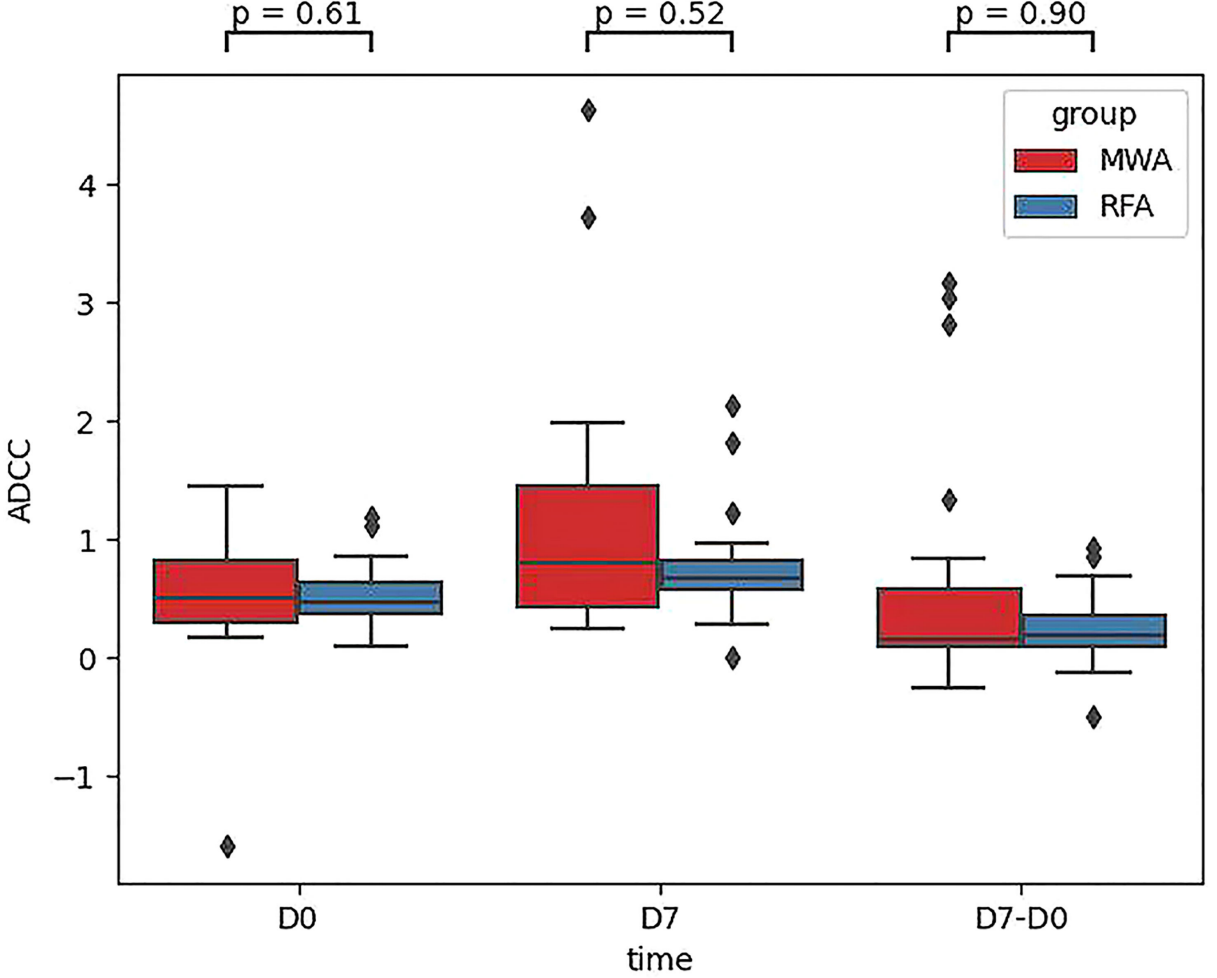
Comparison of the NK cell lysis activity of the target K562 cells between the RFA and WMA groups showed that there was no difference in D0, D7, D7-D0.

**Figure 8:**
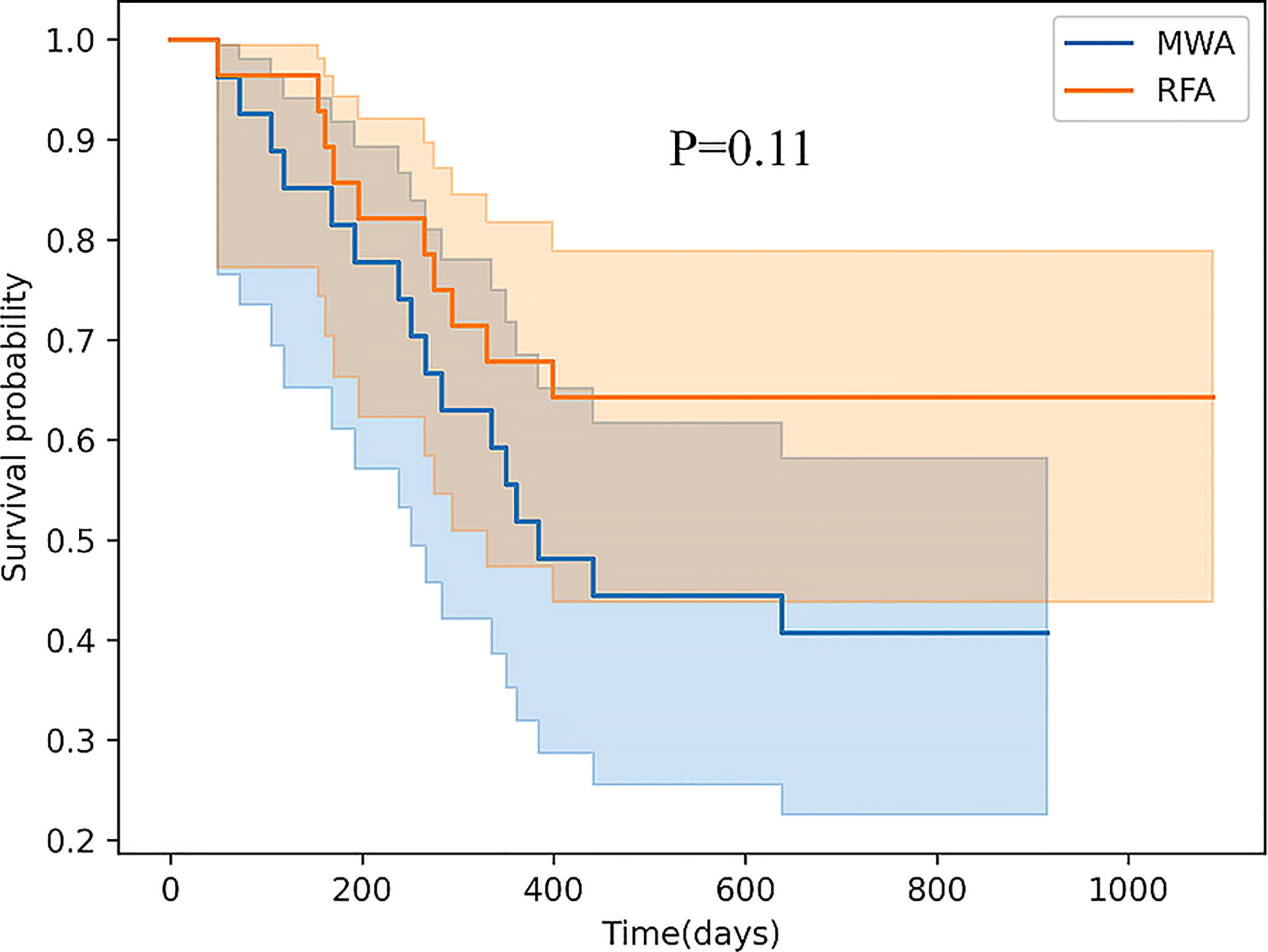
There was no difference in recurrence-free survival (RFS) between the RFA and WMA groups (P=0.11). The median survival time was 361 days for MWA and 460 days for RFA.

Finally, comparing CD107a results between the RFA and WMA groups (D7-D0) suggested that the changes induced by the MWA group were stronger (**Figure 6**). After comparing NK cell lysis activity between the RFA and WMA groups, we found that there was no difference in D0(P=0.61), D7(p=0.52), and D7-D0(p=0.90) (**Figure 7**). No differences in recurrence-free survival were found between the RFA and WMA groups (P=0.51). The median survival time was 361 days for MWA and 460 days for RFA (**Figure 8**).

## Discussion

Because of the positive results in the evaluation of primary hepatocellular carcinoma, several studies have begun to test the combination of an immune syndrome (and other types of immunotherapy) with topical therapy. However, many questions in this area remain to be adressed, including mechanisms, sequence, timing, optimal sites for local therapy, and and optimal immune regulation or immunomodulation. The precise and unique immune effects of topical therapy are not yet known. What kind of treatment is more immune? When is the best time to protect your immune system? The immunogenic effects of local thermal ablation are primarily described in the context of radio frequency ablation (RFA). Only a few preclinical studies have investigated the immune response to microwave ablation (MWA) in liver tissue (20). However, studies comparing the changes in circulating NK cells induced by these two thermal ablation methods have not been conducted.

In this study, a prospective, parallel, controlled experiment was performed to compare the dynamic changes in peripheral blood NK cell subsets, receptors, and functions induced by RFA and MWA and the differences in NK cell immunoregulation induced by RFA and MWA. Our data showed that the frequency of CD3-CD56+ and CD3-CD56+CD16+ in circulating NK cells in peripheral blood was not different in the RFA and WMA groups in D0, D7, M1, D7-D0, M1-D0, and M1-D7 groups. The abundance of NK cell-activated receptors in peripheral blood (NKG2D, NKp46, and NKp30) in the WMA group and the RFA group was compared, and significant differences were found in NK cell-activated receptors (NKG2D, NKp30) at D0, we compared the abundance of the two activated receptors in D7, M1, D7-D0, D7-M1 and M1-D0, indicating that the changes of NKG2D in NK cells induced by RFA were different from those induced by MWA at 1 week (P=0.054). The changes induced by the MWA group were stronger but did not reach significant differences.

In D0, D7, M1, D7-D0, M1-D0 and M1-D7, the frequencies of NK cell inhibitory receptors CD158a and CD158b were not significantly different between the RFA and MWA groups. At D7, the changes of NK cells NKG2A (CD159a) induced by RFA were different from those induced by MWA, and the changes induced by the MWA group were stronger and had a significant difference. Moreover, the frequencies of granzyme B and perforin in peripheral blood NK cells of WMA and RFA groups showed no difference in the D0, D7, M1, D7-D0, M1-D0 and M1-D7 groups. After analysing the IFN-γ induced by the RFA and WMA groups (D7-D0) after a week, we detected that the changes of NK cells induced by RFA were different from those induced by MWA. The changes induced by the RFA group were stronger, but no significant differences were reached. Regarding CD107a in the RFA and WMA groups, (D7-D0) suggested that NK cells were induced to produce CD107a and there was a significant difference between the two groups. In particular, the changes induced by the MWA group were stronger.

To further verify the difference in NK cell killing function induced by RFA and WMA groups, Comparison of the NK cell lysis activity of the target K562 cells between the RFA and WMA groups showed that there was no difference in D0, D7, D7-D0. Analysis of survival showed that There was no difference in recurrence-free survival (RFS) between the RFA and WMA groups.

Our study showed that the differences in MWA and RFA-induced NK cell changes were mainly manifested in the inhibitory receptors CD159a and CD107a one week after surgery in patients with HBV-associated primary liver cancer. CD159a, also known as NKG2A or KLRC1, is a type II transmembrane protein. It belongs to the family of killer cell lectin-like receptors (NKG2 family) and is expressed in NK and NKT cells and T-cell subpopulations. In addition, NKG2A is an inhibitory NK cell receptor that is expressed as a heterodimer with CD94. In humans, NKG2A/CD94 has complex interactions with HLA-E on target cells and inhibits T cell and NK cell effector function. Recent studies have shown that NKG2A blockers enhance the antitumor effects of NK and CD8+ T cells (21, 22). NKG2A blocks NK activation through tyrosine-based immunoreceptor inhibitory motifs in the cytoplasm (23). Previous studies have shown that the inhibitory receptor NKG2A has a high affinity for ligands, which may be related to the assumption that the inhibitory signal is superior to the activation signal (24–26). Even in the presence of inhibitory signals, tumour cells can bind to killer cell activator receptors (KAR) through overexpression of surface antigens, and stress ligands induced by activation of NK cell receptors. When the activation signal exceeds the inhibitory signal, the NK cells are subsequently stimulated to kill the target cell. Lysosome-associated membrane protein-1 (LAMP-1 or CDl07a) is a highly glycosylated protein that accounts for about 50% of all lysosome membrane proteins. When an NK cell kills a target cell, toxic particles reach the surface of the serous membrane and fuse with the cell membrane, causing the release of particle contents and ultimately the death of the target cell. In particular, the upregulation of CD107a was detected as consistent with the secretion of perforin. Therefore, NK cells with CD107a positive expression may have cytotoxic activity (27, 28). Normally, NK cells produce and dominate inhibitory signals to ensure that their own tissue cells are not destroyed, preventing NK from killing healthy cells. When inhibition signals are downregulated or eliminated, NK is activated and releases soluble particles for a killing effect. Unlike adaptive T and B lymphocytes, NK cells do not rearrange their receptor genes on somatic cells but rely on a fixed number of NK cell receptors to inhibit and activate their respective ligands.

Our study showed that microwave and radiofrequency ablation affected the inhibitory NKG2A (CD159a) and activating NKG2D receptors of NK cells one week after the surgery, and also led to changes in NK cell function, including IFN-γ and CD107a. Although we further verified differences between RFA and WMA groups in inducing the NK cell killing function, after comparing the NK cell lysis activity of the target K562 cells in the RFA and WMA groups, we found no difference in D0, D7, D7- D0. Survival analysis showed that these differences did not affect recurrence-free survival (RFS) in the two groups. However, based on the above differences, we suggest combining local treatment with immune checkpoint blockade. In addition, future research is needed to understand what causes these differences. Additionally, more studies are required to elucidate the immune changes of NK cells induced by radiofrequency and microwave ablation, and how NK cell receptors inhibit and activate their respective ligand pathways and signal transduction.

This study has some limitations. First, the sample size for M1 was small. Because of COVID-19, many people can only be tracked locally, resulting in a smaller M1 sample size. Second, since blood only was utilized as our biomaterial, it is impossible to determine whether the observed changes are due to tumour invasion or simple hemodynamics. Third, the limitation of sample size can lead to bias in the data.

## Conclusion

This research aimed to compare the differences between radiofrequency ablation and microwave ablation in inducing the immune regulation of NK cells. We performed comparisons of NK cell subsets, receptors and functions induced by radiofrequency ablation and microwave ablation for HBV-associated primary hepatocellular carcinoma. Our study showed that the differences in NK cell changes induced by MWA and RFA was mainly manifested in the inhibitory receptors CD159a and CD107a one week after surgery in patients with HBV-associated primary liver cancer. To further verify the differences in NK cell killing function induced by RFA and WMA groups, we contrasted the NK cell lysis activity of the target K562 cells in the RFA and WMA groups, and observed no differences in D0, D7, D7-D0. Survival analysis showed that these differences did not affect recurrence-free survival (RFS) in either group of patients.

## Data availability statement

The original contributions presented in the study are included in the article/supplementary material. Further inquiries can be directed to the corresponding author.

## Ethics statement

The studies involving human participants were reviewed and approved by Beijing Youan Hospital. The patients/participants provided their written informed consent to participate in this study.

## Author contributions

Z-PD and H-YW conceptualized the study and prepared figures and tables. H-YW and X-WC wrote the article and prepared figures and tables. Y-HZ and YC collected the data, carried out the analyses, and prepared the figures and tables. N-NL, X-ZY, and S-PS participated in drafting and editing the article and assisted in the preparation of figures and tables. All authors contributed to the article and approved the submitted version.
